# Halogen bonds in some dihalogenated phenols: applications to crystal engineering

**DOI:** 10.1107/S2052252513025657

**Published:** 2013-10-18

**Authors:** Arijit Mukherjee, Gautam R. Desiraju

**Affiliations:** a Solid State and Structural Chemistry Unit, Indian Institute of Science, C. V. Raman Avenue, Bangalore 560 012, India

**Keywords:** crystal engineering, crystal structure prediction, elastic deformation, intermolecular interaction

## Abstract

3,4-Dichlorophenol (**1**) crystallizes in the tetragonal space group *I*4_1_/*a* with a short axis of 3.7926 (9) Å. The structure is unique in that both type I and type II Cl⋯Cl interactions are present, these contact types being distinguished by the angle ranges of the respective C—Cl⋯Cl angles. The present study shows that these two types of contacts are utterly different. The crystal structures of 4-bromo-3-chlorophenol (**2**) and 3-bromo-4-chlorophenol (**3**) have been determined. The crystal structure of (**2**) is isomorphous to that of (**1**) with the Br atom in the 4-position participating in a type II interaction. However, the monoclinic *P*2_1_/*c* packing of compound (**3**) is different; while the structure still has O—H⋯O hydrogen bonds, the tetramer O—H⋯O synthon seen in (**1**) and (**2**) is not seen. Rather than a type I Br⋯Br interaction which would have been mandated if (**3**) were isomorphous to (**1**) and (**2**), Br forms a Br⋯O contact wherein its electrophilic character is clearly evident. Crystal structures of the related compounds 4-chloro-3-iodophenol (**4**) and 3,5-dibromophenol (**5**) were also determined. A computational survey of the structural landscape was undertaken for (**1**), (**2**) and (**3**), using a crystal structure prediction protocol in space groups *P*2_1_/*c* and *I*4_1_/*a* with the COMPASS26 force field. While both tetragonal and monoclinic structures are energetically reasonable for all compounds, the fact that (**3**) takes the latter structure indicates that Br prefers type II over type I contacts. In order to differentiate further between type I and type II halogen contacts, which being chemically distinct are expected to have different distance fall-off properties, a variable-temperature crystallography study was performed on compounds (**1**), (**2**) and (**4**). Length variations with temperature are greater for type II contacts compared with type I. The type II Br⋯Br interaction in (**2**) is stronger than the corresponding type II Cl⋯Cl interaction in (**1**), leading to elastic bending of the former upon application of mechanical stress, which contrasts with the plastic deformation of (**1**). The observation of elastic deformation in (**2**) is noteworthy; in that it finds an explanation based on the strengths of the respective halogen bonds, it could also be taken as a good starting model for future property design. Cl/Br isostructurality is studied with the Cambridge Structural Database and it is indicated that this isostructurality is based on shape and size similarity of Cl and Br, rather than arising from any chemical resemblance.

## Abstract 2

The preference of Br to form type II contacts over type I is explored by various techniques. The mechanical properties of some dihalogenated phenols are correlated with their structures.

## 1. Introduction   

A halogen bond *R*—*X*⋯*Y*—*Z* occurs when there is evidence of a net attractive interaction between an electrophilic region on a halogen atom *X* belonging to a molecule or a molecular fragment *R*—*X* (where *R* can be another atom, including *X*, or a group of atoms) and a nucleophilic region of a molecule, or molecular fragment, *Y*—*Z* (Desiraju *et al.*, 2013 ▶). Over the years, the halogen bond has been discussed in several contexts in structural chemistry (Metrangolo & Resnati, 2001 ▶). In recent times, it has entered the literature of crystal engineering and has been used in crystal design strategies (Metrangolo *et al.*, 2005 ▶). Halogen bonding has been traditionally monitored with spectroscopic (Harris *et al.*, 1974 ▶), computational (Price *et al.*, 1994 ▶) and crystallographic techniques (Bent, 1968 ▶). Typically, simpler systems have been studied with spectroscopy, as for example the molecular beam experiments on the Cl_2_⋯Cl_2_ dimer (Janda *et al.*, 1976 ▶), which has implications for crystallization mechanisms, as do studies of the halogen bond in solution (Erdélyi, 2012 ▶; Mukherjee & Desiraju, 2011 ▶). As in hydrogen bonding, more complex systems are better studied with crystallography. In this case, halogen bonding is usually monitored in terms of shortness of the contacts between halogen atoms and the nucleophile and their angles of approach. In the halogen bonds studied here, the nucleophile is mostly halogen, in other words, we are referring to contacts of the type *X* ^δ+^⋯*X* ^δ−^, although it should be noted that it is only the electrophilic halogen *X* ^δ+^ that renders the name ‘halogen bond’ possible (Glaser *et al.*, 2006 ▶; Metrangolo *et al.*, 2006 ▶). This invokes two possibilities for the formation of halogen bonds. Firstly, and as proposed by Williams & Hsu (1985 ▶), there can be an attractive interaction between the halogen atoms and this assumption gets support from various earlier studies, such as the preference of the orthorhombic *Cmca* structure over the isotropic cubic *Pa*3 structure for the Cl_2_ crystal (Collin, 1952 ▶) and even the very existence of a Cl_2_⋯Cl_2_ dimer. Alternatively, and as per the findings of Nyburg, there can be decreased repulsion between the two halogen atoms in *X*⋯*X* because of the non-spherical distribution of the atomic charge density (Nyburg & Wong-Ng, 1979*a* ▶,*b* ▶); this too finds support from experimental charge density analysis on crystalline Cl_2_ which shows the absence of any density peaks between nearest neighbour molecules (Stevens, 1979 ▶; Burgos *et al.*, 1982 ▶). The study of the interplay between these two models (electrostatics as a chemical model and anisotropy as a geometrical model) is more usefully carried out with halogen bonds compared with hydrogen bonds, because the sizes of the halogen atoms are significantly larger than that of the H atom. In an early work, Sakurai, Sundaralingam and Jeffrey noted that halogen⋯halogen contacts, say Cl⋯Cl, are of two types: (*a*) both C—Cl⋯Cl angles are equal and around 160 ± 10° or (*b*) one of the angles is close to 175° and the other is around 80° (Sakurai *et al.*, 1963 ▶). It may be noted that the first situation is compatible with a centre of inversion and is typical of (although obviously not exclusive to) triclinic space groups, whereas the latter is observed mostly in monoclinic and orthorhombic space groups, being compatible with screw and glide symmetry. More than two decades ago, Desiraju and Parthasarathy[Chem scheme1] used statistical analysis and classified halogen⋯halogen contacts into two major categories, namely type I (θ_1_ = θ_2_) and type II (θ_1_ = 180, θ_2_ = 90) where θ_1_ and θ_2_ are the two C—Cl⋯Cl angles (Desiraju & Parthasarathy, 1989 ▶). The type I/type II nomenclature seems to have been generally accepted.

The type I contact is considered to be van der Waals in nature because the symmetrical approach of halogen atoms is incompatible with the electrophile–nucleophile character of a true halogen bond. An electrostatic explanation for type I contacts in a certain angle range has been provided (Awwadi *et al.*, 2006 ▶). However, if this were completely true, the proportion of type I to type II contacts for Cl⋯Cl, Br⋯Br and 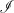
⋯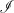 contacts would not be as different from one another as is observed in reality (type I being more common for Cl and type II being more common for 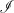). In this, and in the rest of the paper, the symbol for the element iodine is given as ‘
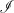
’ to distinguish it from the symbol ‘I’ which is used to denote ‘type I’. The type II contact involves an approach of the electrophilic region of one halogen atom with the diffuse electron density of the other (Bui *et al.*, 2009 ▶). Accordingly, it qualifies as a true halogen bond according to the modern definition. It is of importance therefore to distinguish properly between type I and type II *X*⋯*X* contacts (Tothadi *et al.*, 2013 ▶). Chemically speaking, they are quite different. This paper provides confirmation, with respect to the title dihalogenated phenols, that this is indeed the case.
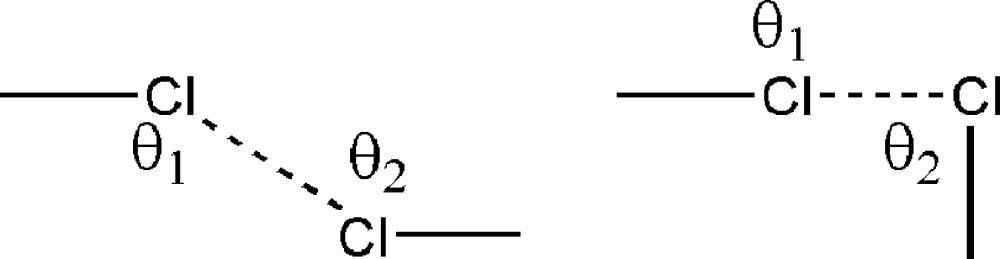


Among the halogens, Cl, Br and 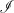 stand out as potential halogen-bond formers because of the presence of an electrophilic region in the charge distribution of the covalently bonded atom in say C—*X*. This behaviour is very different from F and the argument finds experimental support in the fact that fluorine crystallizes in the (isotropic) cubic *Pm*3*n* structure, whereas all the other halogens adopt an anisotropic layered orthorhombic structure in space group *Cmca*. These layered crystal structures could be a result of shape anisotropy (Nyburg model) and/or polarizability (Williams model) in the three heavier halogens. The situation is more complicated in that while polarizability is more important for 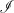, anisotropy is significant for Cl. The study carried out by Miller, Paul and Curtin on halogen-substituted acids and anhydrides showed that in 4-chlorobenzoic acid, acid dimers are connected through type I Cl⋯Cl contacts, whereas in 4-iodobenzoic acid, 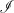
⋯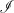 contacts are type II (Miller *et al.*, 1974 ▶; Patil *et al.*, 1985 ▶). This early observation indicates that the likelihood of formation of type II contacts increases from Cl to 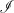. Being positioned between these two extremes, Br is more difficult to interpret and understand. The slightly shorter Br⋯Br contact (3.203 Å) in triphenylbromomethane (Dunand & Gerdil, 1984 ▶) when compared with the corresponding Cl⋯Cl contact (3.21 Å) in the isomorphous triphenylchloromethane (Dunand & Gerdil, 1982 ▶) indicates that Br⋯Br is stronger than Cl⋯Cl and that Br is more polarizable than Cl. Ample evidence of type II Br⋯Br synthons, say Br_3_ synthons, in crystal engineering also confirms the polarizable nature of Br (Bosch & Barnes, 2002 ▶). On the other hand, there exists the Cl/Br exchange rule which acknowledges the similarity between Cl and Br in many structures. Therefore, even after much exploitation of halogen bonds in crystal engineering, the nature of Br remains blurred and any study to this end would be useful to the future application of halogen bonds.

While the identification of short halogen⋯halogen contacts in organic crystal structures and their analysis can be traced back several decades (Hassel, 1970 ▶) and there has been little doubt regarding their role in crystal structures (for a typical example, see Freytag *et al.*, 1999 ▶), these contacts have only come into mainstream crystal engineering after Resnati and Metrangolo coined the term *halogen bond* taking into account their similarity with hydrogen bonds (Metrangolo & Resnati, 2001 ▶; Metrangolo *et al.*, 2005 ▶; Metrangolo, Resnati *et al.*, 2008 ▶; Metrangolo, Meyer *et al.*, 2008 ▶; Rissanen, 2008 ▶; Fourmigué, 2009 ▶; Politzer *et al.*, 2010 ▶; Legon, 2010 ▶). Earlier applications include the steering nature of halogens towards 4 Å short-axis structures (β-structure; Sarma & Desiraju, 1986 ▶) and thereafter controlling solid-state reactivity (Green & Schmidt, 1970 ▶). Their use in coordination chemistry and the exploration of metal–halogen bonds as halogen-bond acceptors (Zordan *et al.*, 2005 ▶; Brammer *et al.*, 2008 ▶) is documented. However, it is Resnati and Metrangolo who demonstrated the great potential of the halogen bond in logic-derived crystal engineering, based on the supramolecular synthon (Metrangolo *et al.*, 2008 ▶). Subsequently, there have been several applications of halogen bonds in design strategies (Aakeröy *et al.*, 2007 ▶), for example as an element for structural insulation, among others. Taking all this into account, there have still been very few studies which address the relative behaviour of halogens in terms of type I and type II contacts and which correlate the properties that emerge therefrom. It is only recently that halogen bonds have been shown to be useful in tuning properties and therefore a promising area of future research in crystal engineering (Reddy, Kirchner *et al.*, 2006 ▶; Yan *et al.*, 2011 ▶). While some crystal properties have been correlated with halogen bonds, systematic studies with respect to mechanical properties are still missing, to the best of our knowledge.

In this context, we started this study to address three different but related issues: (1) to investigate the nature and preference of halogen bonds formed by Br, using a technique of alternative chemical substitution in phenol (**1**); (2) to distinguish between type I and type II halogen⋯halogen contacts experimentally; (3) to correlate mechanical properties with halogen-bonding characteristics. Cambridge Structural Database (CSD) analyses and computational surveys of the structural landscape have been carried out in parallel, which complement and assist our experimental findings.

## 2. Experimental   

### 2.1. Materials   

Phenols (**1**), (**2**), (**4**) and (**5**) were purchased from Aldrich, Alfa Aesar or Sigma Aldrich, and used without further purification. (**3**) was synthesized using a reported procedure (Bosmans *et al.*, 2009 ▶).

### 2.2. Crystallization   

Polymorphism was checked for all the compounds using rigorous protocols that are customary in our group. A large number of crystallizations are carried out in different conditions (sublimation, crystallization) and with various solvents and solvent mixtures. The phenols generally crystallize well if *n*-hexane is taken as a non-solvent. PXRD was routinely recorded for the solids in the crystallization vessel and were matched with simulated patterns generated from respective single-crystal data. At least five or six single crystals were examined on the diffractometer for each phenol. No polymorph was found for any of the compounds under these conditions.

#### 2.2.1. 3,4-Dichlorophenol (**1**)   

The compound was crystallized from both MeOH/*n*-hexane and CHCl_3_/*n*-hexane solvent mixtures. The minimum amount of the solvent was added to dissolve the compound that was taken initially in a small quantity of the non-solvent. The crystallization was tried in a cooling oven at 252 K and also at room temperature. Very thin crystals were obtained after 3–4 d under any of these conditions.

#### 2.2.2. 4-Bromo-3-chlorophenol (**2**)   

This compound was crystallized from CHCl_3_/*n*-hexane solvent mixture. The crystallization was performed in a cooling oven at 252 K. Very thin crystals were obtained after 3–4 d.

#### 2.2.3. 3-Bromo-4-chlorophenol (**3**)   

The compound was synthesized using the literature procedure, as mentioned above, and crystals were obtained by quenching the product in liquid N_2_.

#### 2.2.4. 4-Chloro-3-iodophenol (**4**)   

This compound was crystallized from MeOH/*n*-hexane solvent mixture. The crystallization was attempted in the refrigerator at 278 K and also at room temperature (298 K). Crystals were obtained after 3–4 d.

#### 2.2.5. 3,5-Dibromophenol (**5**)   

The compound was crystallized from MeOH/*n*-hexane solvent mixture. The crystallization was carried out at room temperature. Crystals were obtained after 3–4 d.

### 2.3. Single-crystal X-ray diffraction   

Single-crystal X-ray data were collected on a Rigaku Mercury375R/M CCD (XtaLAB mini) diffractometer using graphite-monochromated Mo *K*α radiation, equipped with a Rigaku low-temperature gas-spray cooler. Data were processed with the Rigaku *CrystalClear* software (Rigaku, 2009 ▶). Structure solution and refinements were performed using *SHELX*97 (Sheldrick, 2008 ▶) within the *WinGX* suite (Farrugia, 1999 ▶). For (**1**) and (**2**), data were collected after mounting the crystals inside a glass capillary. For (**1**), (**2**) and (**4**), data were collected at three different temperatures (150, 200, 296 K) for the variable-temperature study. The datasets for (**3**) and (**5**) were collected at 150 K. Although the diffraction patterns and the spot shapes appear to be acceptable, the s.u.s on some of the unit-cell parameters are (reproducibly) high (Table 1 ▶), and we do not currently have any reasonable explanation for this.

### 2.4. Crystal structure prediction (CSP)   

A CSP protocol was applied to compounds (**1**), (**2**) and (**3**) in order to provide an (coarse) overview of their crystal structure landscape. The molecular structure of (**1**) extracted from its crystal structure (at 150 K) was taken initially. For (**2**) and (**3**), halogen substitutions were made in the respective positions in (**1**). Each molecule was then optimized using *DMol* ^3^ (Delley, 1990 ▶) and ESP charges (electrostatic potential energy surface derived charges) were assigned after the optimization. The charge-assigned optimized structure was taken as an input for the CSP using the *Polymorph Predictor* module in *Materials Studio* (Accelrys, 2011 ▶), with the COMPASS26 force field (Sun, 1998 ▶). The search was restricted to space groups *P*2_1_/*c* and *I*4_1_/*a* for all three compounds. After completion of the calculation, the top 100 structures were taken, based on the lowest total energy and then on the highest density. The detailed procedure of the CSP protocol is provided in the supporting information.

### 2.5. CSD study: analysis of Cl/Br isostructurality   

A list of refcodes was obtained from the CCDC, upon request, of 1867 organic and organometallic compound pairs wherein both chloro and bromo analogues are present in the database. Effectively, these are pairs of molecules where the only difference is that at least one C—Cl bond in one is replaced by a corresponding C—Br bond in the other. The data were further processed by us to obtain 1127 pairs of C—Cl and C—Br compounds in which the reduced cell edges differ by less than 1 Å. These pairs of compounds were examined manually and 1060 pairs of crystals were found where the space groups and *Z* values are the same. In most of these pairs, there are no Cl⋯Cl (or Br⋯Br) interactions. In 152 pairs, however, such interactions were identified up to a limit of the van der Waals distance plus 0.2 Å. In a subset of these 152 pairs, there are 95 pairs in which a Cl⋯Cl interaction in one of the structures is replaced by a Br⋯Br interaction in the other, with practically no other significant difference. The remaining 57 pairs include those with Cl/Br disorder (9 pairs), structures which cannot be properly classified as type I or type II (16 pairs), pairs which have Cl⋯Br interactions (27 pairs) and pairs wherein one molecule contains one Cl atom and one Br atom and with two halogen interactions: the first is a Cl⋯Cl (or Br⋯Br) interaction within the prescribed distance limit and the second is a long Cl⋯*X* (or Br⋯*X*) interaction (5 pairs; data from such structure pairs would be inconclusive). The 95 pairs of compounds are given in the supporting information and were divided further according to whether the Cl⋯Cl (and Br⋯Br) contacts are type I or type II using criteria that were recently suggested (Tothadi *et al.*, 2013 ▶).

## 3. Results and discussion   

### 3.1. 3,4-Dichlorophenol as a model compound in an alternative substitution strategy   

**Figure 1:**
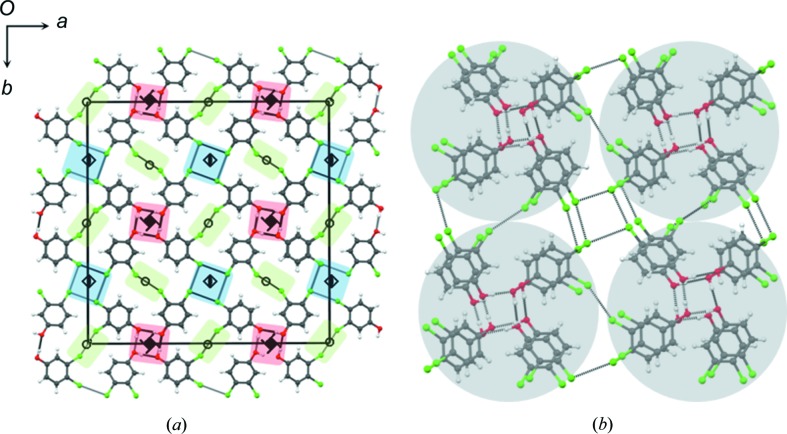
(*a*) Crystal structure of 3,4-dichlorophenol: hydrogen bond (red), type I contacts (green) and type II contacts (blue) showing the symmetry requirement for different non-covalent interactions. (*b*) The halogen bond acts as interlayer glue pulling two layers together.

3,4-Dichlorophenol (**1**) (Bavoux *et al.*, 1980 ▶) crystallizes in the tetragonal space group *I*4_1_/*a*, with a short axis of 3.7926 (9) Å (Fig. 1 ▶ *a*). The O—H⋯O hydrogen bond is the strongest interaction possible in this structure and it propagates around the 4_1_ screw axis. Generally, it is not expected that a chlorophenol adopts a 4 Å structure (β-structure) because the optimization of O—H⋯O bonds in such a packing could preclude the Cl⋯Cl interactions. For instance, hydrogen bonding around a 2_1_ screw axis of around 4.8 Å is typical for phenols and some extra stabilization is obtained from weaker interactions which include Cl⋯Cl. The importance of Cl⋯Cl contacts increases as the number of Cl atoms in the molecule increases. A study on the six isomeric dichlorophenols by Thomas & Desiraju (1984 ▶) showed why 2,3-, 2,4- and 3,4-dichlorophenols adopt β-structures, while the three other isomeric variants adopt non-β structures. The β-structures are further associated with higher symmetries (trigonal and tetragonal). These higher-fold 3_1_ and 4_1_ axes are possible because the substitution pattern does not result in bad contacts even with a β-structure, and indeed the 4 Å packing goes hand in hand with the higher symmetry (Desiraju, 2004 ▶). If the three other isomers take high-symmetry β-structures, bad contacts would arise, and therefore they are monoclinic and do not have the β-structure. Among the β-compounds, 3,4-dichlorophenol is tetragonal rather than trigonal because the chloro substitution is in the molecular periphery. The adoption of the 4_1_ axis by 3,4-dichlorophenol may therefore be attributed to two factors: (i) substitution of the Cl-groups in the 3- and 4-positions; (ii) Cl⋯Cl contacts made with the neighbouring molecules. Fig. 1 ▶(*b*) shows both these features. Halogen bonding plays a major role in this structure. The adoption of the tetragonal β-structure is a result of the positional compatibility of the OH and Cl substituents in the molecule.

What is pertinent to the present study is that the 3,4-dichlorophenol crystal structure contains both type I and type II Cl⋯Cl contacts which are roughly perpendicular to the unique axis. The 3-chloro group makes a type I contact across an inversion centre [3.235 (1) Å] and the 4-chloro group makes a type II contact [3.408 (1) Å] that relates the Cl atoms with a 4 axis. This is a very rare phenomenon. Not only are type I and type II Cl⋯Cl contacts chemically distinct, they are also weak. The predominance of strong interactions (like O—H⋯O) often suppresses the subtle differences in the weak interactions. This makes the simultaneous occurrence of type I and type II contacts in a hydrogen-bonded compound the rarest of situations: (**1**) is the only structure to the best of our knowledge which exhibits this feature. The type I contact distance is in the limit of the repulsive region, while the type II contact is attractive. Both contacts are interstack rather than intrastack (Fig. 1 ▶). The helical stacks of 3,4-dichlorophenol can be considered as having a hydrophilic core (O—H⋯O) and a hydrophobic exterior (Cl⋯Cl). The relevance of halogen bonding in the organization of the structure and simultaneous presence of both types of Cl⋯Cl contacts makes (**1**) suitable as a model compound in our alternative substitution strategy.

As type I and type II are chemically different in nature, the crystal structure of 3,4-dichloro­phenol, in which these two contacts are evenly balanced, lends itself well to a calibration of type I and type II Cl⋯Cl and Br⋯Br contacts/interactions. We therefore posed to ourselves the following questions: (i) what would happen if the Cl atom in the 4-position is replaced with a Br atom, leaving the Cl atom in the 3-position unchanged? (ii) Likewise, what would happen if the Cl atom in the 3-position is replaced with a Br atom, leaving the Cl atom in the 4-position unchanged? The hypothesis is that these two substitution changes would not have the same consequence, if chemical effects are important. If Br has a similar geometrical effect on crystal packing as Cl (as expected from Cl/Br exchange in many structures where Cl⋯Cl is important), then both the structures should be isostructural to the model compound. Accordingly, we determined the crystal structures of 4-bromo-3-chlorophenol (**2**) and 3-bromo-4-chlorophenol (**3**).

**Figure 2:**
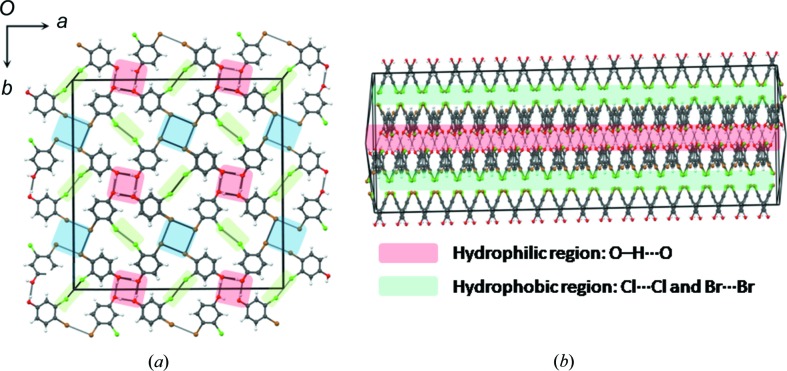
4-Bromo-3-chlorophenol (**2**): (*a*) Hydrogen bond and halogen bond pattern, O—H⋯O (red), type II Br⋯Br (blue) and type I Cl⋯Cl (green). (*b*) Calculated Bravais–Friedel–Donnay–Harker (BFDH) morphology showing hydrophilic core and hydrophobic exterior. This makes the structure anisotropic in orthogonal directions. Colour code: hydrogen bond region (red), halogen bond region (cyan).

The crystal structure of (**2**) is isomorphous to that of (**1**) with Br in the 4-position participating in a type II interaction [3.5379 (7) Å], and Cl in the 3-position with a type I interaction [3.241 (1) Å] similar to that in (**1**). Therefore, in (**2**), very much like (**1**), there is the presence of a hydrophilic interior core (dominated by O—H⋯O) and a hydrophobic exterior (dominated by Br⋯Br and Cl⋯Cl) and as a result the structure becomes highly anisotropic between the direction of the tetragonal short axis and other orthogonal directions (Fig. 2 ▶). Moreover, the Br⋯Br distance is much shortened in this structure in comparison to the type I Cl⋯Cl contact in (**1**). We conclude that type II Br⋯Br and type I Cl⋯Cl interactions are both justified and compatible within the overall framework of this tetragonal structure.

**Figure 3:**
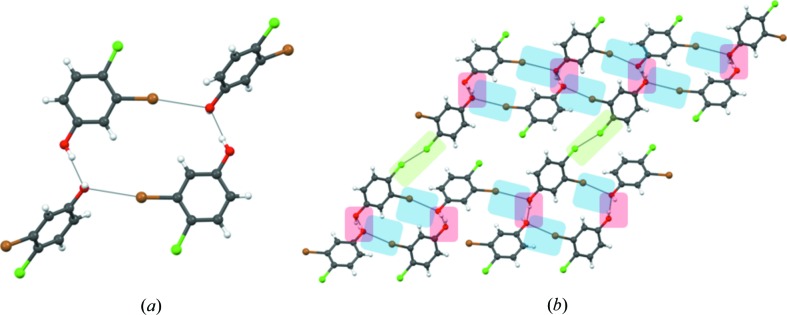
3-Bromo-4-chlorophenol (**3**): (*a*) Primary synthon comprising O—H⋯O hydrogen bonds and short Br⋯O interactions. (*b*) Primary synthons joined through Cl⋯Cl type I contacts. Colour code: O—H⋯O hydrogen bond (red), type II Br⋯O (blue) and type I Cl⋯Cl (light green).

The test case is compound (**3**) in which Br is located in the 3-position and Cl is in the 4-position. Here, the packing is quite different (*P*2_1_/*c*) and while the structure is still sustained through O—H⋯O hydrogen bonds, the tetramer synthon in (**1**) and (**2**) is not observed. There is no type I Br⋯Br interaction. Br still prefers a type II contact with the alternative nucleophile, oxygen. The Br⋯O contact [3.029 (2) Å] is very short. Even more tellingly, the Cl atom placed in the 4-position prefers to form a type I interaction, unlike its behaviour in compound (**1**). In effect, the tendencies shown by the halogen atoms in (**1**) are reversed in compound (**3**) (Fig. 3 ▶). Rather than being dictated to by their position in the molecule, Cl and Br behave according to their chemical nature: Cl prefers type I and Br prefers type II. It requires just a small chemical perturbation (3-Cl → 3-Br) to upset the structure of (**1**) completely.

**Figure 4:**
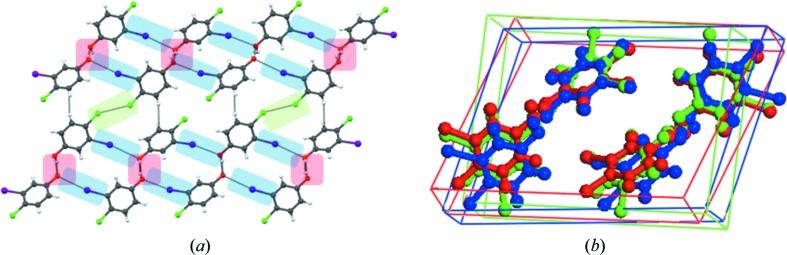
4-Chloro-3-iodophenol (**4**): (*a*) Packing diagram with O—H⋯O hydrogen bonds (red), type II 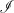
⋯O (blue) and type I Cl⋯Cl (light green) interactions. (*b*) Structural overlap between (**3**) (red), (**4**) (blue) and (**5**) (green).

To assess the generality of our rationalization, 4-chloro-3-iodophenol (**4**) was studied next. Compound (**4**) shows similar hydrogen and halogen bonding synthons [Cl⋯Cl, 3.414 (5) Å; 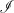
⋯O, 3.162 (6) Å] and may be compared directly with (**3**); it is actually isomorphous with (**3**) (Fig. 4 ▶), and it is a case of Br/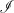 isostructurality. To get an idea about the structural class of both (**3**) and (**4**), we also determined the crystal structure of 3,5-dibromophenol (**5**) (shown in S6 of the supporting information). The similarity between structures (**3**), (**4**) and (**5**) is instructive from the overlap diagram (Fig. 4 ▶ *b*). One can conclude that in (**1**), both type I and type II Cl⋯Cl contacts are sustainable within tetragonal symmetry. Phenol (**2**) is isostructural to (**1**), because the type II contact is strengthened in going from Cl⋯Cl to Br⋯Br, while the type I contact is unchanged. Compound (**3**) takes a different structure because, if the same structure were retained, the type I contact would have been weakened by going from Cl⋯Cl to Br⋯Br with the type II Cl⋯Cl contact remaining unchanged. Therefore, the introduction of Br in the 3-position of (**1**) changes the structure from a β- to a non-β-structure. Phenols (**2**) and (**3**) are not only very different in the formation of their primary synthons but they also belong to two very different structural classes. The structures of (**3**), (**4**) and (**5**) reveal the similarity of Br and 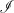 with respect to their propensity to form type II interactions. Thomas & Desiraju (1984 ▶) argued that while (**1**) takes a β-structure, 3,5-dichlorophenol has a non-β-structure, because a β-structure for the latter would have many bad contacts. In the context of the present work, it may be said that (**3**) does not take the structure of (**1**) because of the bad type I Br⋯Br contacts that would have to be formed. All of this again reinforces the observation that Br prefers to form type II over type I (Felsmann *et al.*, 2011 ▶).

### 3.2. Computational study: CSP to locate the structures in the energy landscape   

**Figure 5:**
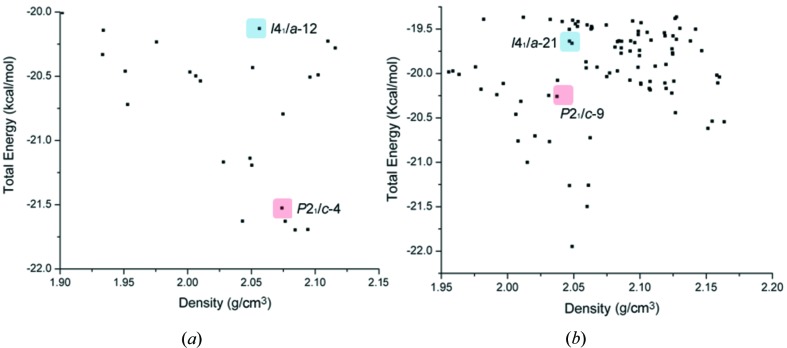
(*a*) Energy landscape for (**2**) with experimental structure of (**2**) highlighted in blue and the nearest match to structure (**3**) in red. (*b*) Energy landscape for (**3**) with the experimental structure of (**3**) highlighted in red and hypothetical structure similar to (**2**) in blue.

In addition to predicting experimental crystal structures from molecular structures (Bardwell *et al.*, 2011 ▶; Kendrick *et al.*, 2011 ▶), CSP is a useful exercise to locate hypothetical structures which are in the same range of energy as the experimental structure, but are not physically observable or experimentally accessible (Dubey *et al.*, 2012 ▶). Even at a coarse level, such an exercise can be useful. In the context of our work, we used CSP to locate the experimental structures for (**2**) and (**3**) within the energy landscape (Price, 2008 ▶) and to examine other structural possibilities for these two compounds. A CSP protocol was performed on (**1**), (**2**) and (**3**) in two space groups (*P*2_1_/*c*, *I*4_1_/*a*) with the COMPASS26 force field after taking the experimental molecular structure of (**1**) as an input. The protocol we used does not consider the anisotropy in halogen atoms (Day & Price, 2003 ▶) and the ranking and location of these structures in the energy landscape is completely based on electrostatics and van der Waals contacts. Therefore, the aim is not to predict the observed crystal structure as the most stable structure in the list, but only to provide a general idea about the structural landscape (Mukherjee *et al.*, 2011 ▶; Tothadi & Desiraju, 2012 ▶). The appearance of the experimental structure of (**1**) in the 15th position in its own landscape is an indication that the force field chosen for the study is adequate and that it can be used in the exploration of landscapes for these three compounds. CSP results are typically evaluated in terms of the overlap of 15 molecules (and the r.m.s. value) in the immediate coordination sphere of a reference molecule with the structure to be compared. In the landscape of (**1**), the experimental structure of (**2**) also appears in the 15th position [(**1**) and (**2**) being isomorphous], but the closest structure to (**3**) appears in the 44th position, although the match is not good. Only nine molecules in the 44th structure match with the experimental structure of (**3**). This result indicates that Cl⋯O is not a preferred interaction in (**1**) and that the combination of type I and type II Cl⋯Cl contacts is preferred. In the landscape of (**2**), the experimental structure of (**2**) appears in the 23rd position with an r.m.s. of 0.339 (Fig. 5 ▶). Although the closest structure to (**3**) appears in the 5th position in this landscape, only 13 out of 15 molecules of the experimental structure match, and that too with a high r.m.s. (0.582). This result shows that (**2**) prefers to adopt the tetragonal structure over the monoclinic one and the reasons for this preference have been detailed in §3.1. Analysis of the other structures in this landscape also shows that there are very few synthon possibilities among them that appear in the experimental structures. The experimental structure of (**3**) appears in the 17th position in the energy landscape of (**3**). Interestingly, the structure of (**2**) appears in the 63rd position in the same landscape. This definitely shows that the change of the Br location from 4-Br to 3-Br is not favourable with respect to the tetragonal structure. This is in line with our experimental observations. The polarizable nature of Br *vis-a-vis* Cl is strongly evident. Assuming that these are the two structural types available for these compounds, the significant differences in the overall features of the landscapes of (**2**) and (**3**) indicate the electrophilic nature of Br and its propensity for the formation of type II contacts. This study shows that when both choices are energetically available, (**3**) prefers to choose the non-β structure indicating that Br prefers to make type II over type I contacts.

### 3.3. Variable temperature crystallography as a means of distinguishing type I and type II halogen⋯halogen contacts   

In order to differentiate experimentally between type I and type II halogen contacts, which are chemically distinct, and have different distance fall-off properties, a variable temperature crystallography study (VT study) was performed on compounds (**1**), (**2**) and (**4**) (Table 2 ▶). There are two earlier reports of variable temperature crystallography on halogen atom contacts (Forni *et al.*, 2003 ▶; Mínguez Espallargas *et al.*, 2008 ▶), but there is no detailed attempt to distinguish type I and type II halogen contacts in these studies. Mínguez Espallargas *et al.* studied the variation of these contacts with high pressure and low temperature and noted that type I contacts are more compressible than type II contacts, but they also pointed out the known fact that the effects of high pressure and low temperature need not be the same. Among the three compounds mentioned above, (**1**) and (**2**) are of central importance. We have already mentioned that type I contacts are of the van der Waals variety whereas type II are electrostatic, and therefore more long-range. Type I contacts are also weaker than type II contacts. Accordingly, it was expected that the type I contacts would show a smaller distance variation with temperature than type II contacts because they are more short range. The Cl⋯Cl and Br⋯Br contacts in (**1**) and (**2**) were accordingly analysed.

The VT study reveals that in both (**1**) and (**2**) the percentage increase in the *X*⋯*X* distances upon raising the temperature is more prominent for type II than type I. In (**1**), the type II Cl⋯Cl contact distance smoothly increases by 1.8%, when the temperature is changed from 150 to 296 K, whereas the type I contact increases by only 1.0%. The electrostatic type II contacts are viable at longer distances and so can lengthen or shorten more easily with temperature variations. Type I contacts are more van der Waals in nature and so do not lengthen and shorten so much when the temperature is varied. The type II Br⋯Br distance in (**2**) shows a wider variation with temperature (2.1%) than the corresponding type II Cl⋯Cl contact in (**1**) (1.8%) because Br is more polarizable than Cl. The internal check on the accuracy of these measurements is that the increase in the distance of the type I Cl⋯Cl contact in both (**1**) and (**2**) is practically the same. The benchmarking of an unambiguous type II interaction is the 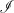
⋯O contact in (**4**). This increases by 1.6%, showing that the Br⋯Br contact in (**2**) (which has a larger increase with temperature) is indubitably type II. The overall increases in the cell volume are unexceptional, but what is more revealing is that different regions of the structure behave slightly differently from one another, and these differences may be correlated with chemical differences. As an added check, in response to a referee comment, we carried out a variable temperature study of the type I Cl⋯Cl contact in the unrelated 4-chlorobenzoic acid (1.1% increase between 150 and 296 K), and of the type II Cl⋯Cl contact in 2,3,5-trichloro­salicylic acid (2.1% increase between 150 and 296 K). In all this, one notes that the type II contact elongates or contracts more with temperature change than the type I contact because of its more flexible distance dependence character, not because it is stronger. Dependence on pressure may have more to do with the latter characteristic. To summarize, the type II contacts are electrostatic in nature and should be considered as true halogen bonds. Further, we have presented a convenient way of experimentally distinguishing between type I and type II halogen contacts.

### 3.4. Application in crystal engineering: correlation of structures with mechanical properties   

Crystal engineering by definition has three steps in its execution: The first is the analysis of crystal structures; the second is the development of a design strategy; the third is the crystal synthesis of a family of structures whose properties are examined leading ultimately into property design (Desiraju, 1989 ▶). The study of mechanical properties in molecular crystals has been a subject of considerable interest for decades (Boldyrev, 1996 ▶). Generally, molecular crystals which have comparable interactions in all directions are brittle in nature (Wright, 1996 ▶). In the low stress region they may display elastic behaviour, as has been recently shown by Ghosh & Reddy (2012 ▶). This is generally true of crystals of molecules of low polarity, which are mostly packed in a herringbone pattern, or alternatively in crystals which are extensively hydrogen-bonded in all directions, for example sugars. On the other hand, plastic bending in molecular crystals occurs when the packing is anisotropic, and strong and weak interactions are oriented in nearly orthogonal directions (Reddy *et al.*, 2005 ▶; Reddy, Padmanabhan *et al.*, 2006 ▶). A bending crystal can be usually bent in just one particular direction and cannot be deformed in any arbitrary way. This type of bending is a manifestation of plastic deformation in a molecular crystal, and is different from bending in metallic crystals in that there is no change in the volume of the crystal after deformation.

**Figure 6:**
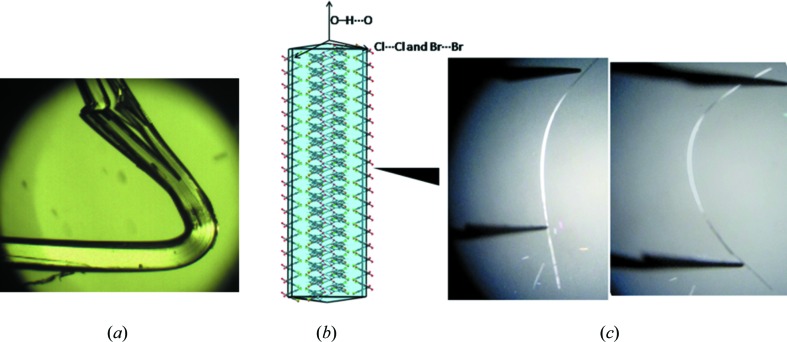
(*a*) Plastic bending observed in (**1**). (*b*) Predicted BFDH morphology for (**2**), (*c*) elastic bending in (**2**).

The tetragonal space group *I*4_1_/*a* of 3,4-dichlorophenol implies that equivalent interactions (in this case either type I or type II) exist along the *a* and *b* directions. Considering that the Cl⋯Cl interactions (weaker than O—H⋯O) are coincident with the (001) plane, the bending direction is not merely [100] and [010] but any combination of these directions. Accordingly, the plastic bending of crystalline (**1**) is highly irregular and convoluted (Fig. 6 ▶ *a*) and this has been explained in an earlier publication (Reddy *et al.*, 2005 ▶). When we attempted to deform crystalline (**2**), we noticed that rather than undergo plastic bending like (**1**), the crystals showed elasticity (see the video in the supporting information). It has been demonstrated that the type II Br⋯Br interaction in (**2**) is stronger than the corresponding Cl⋯Cl interaction in (**1**). The elastic nature of (**2**) is rationalized by the fact that the type II Br⋯Br interaction is comparable enough energetically with the O—H⋯O hydrogen bond so that a degree of interaction isotropy is achieved. The energy difference between a type II Cl⋯Cl and a type II Br⋯Br interaction is great enough to change the mechanical response from plastic to elastic under similar loads. The stronger Br⋯Br interactions operate at longer distances and as a corollary can regain their original position after being deformed (Fig. 6 ▶). The restoring ability of these forces is sufficient to maintain elastic behaviour. Significant elastic deformation is very rare in molecular crystals because of their inherent anisotropic character (Ghosh & Reddy, 2012 ▶). Therefore, the observation of elastic behaviour in (**2**) is noteworthy; in that it finds a possible explanation based on the strengths of the respective halogen bonds, it could also be taken as a good starting model for future property design.

**Figure 7:**
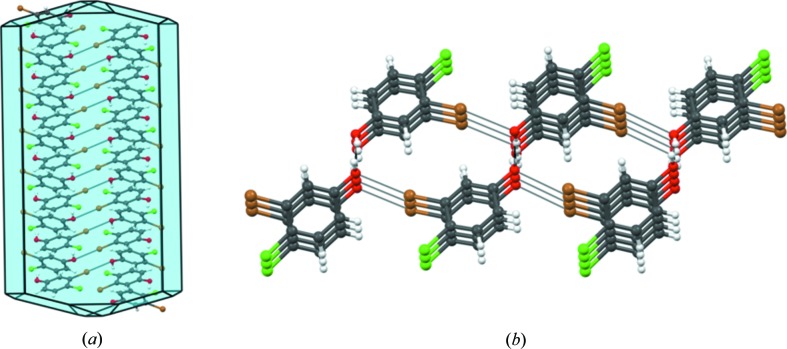
3-Bromo-4-chlorophenol (**3**). (*a*) Predicted BFDH morphology; (*b*) strong O—H⋯O and Br⋯O interactions perpendicular to the short axis.

Crystals of (**3**), (**4**) and (**5**), on the other hand, are brittle. These three structures are not tetragonal, and are sustained by O—H⋯O and Br⋯O or 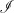
⋯O interactions in all three directions. They do not satisfy the condition of orthogonal anisotropy required for plastic bending. In order to explain the brittle property in (**3**), (**4**) and (**5**), we have taken (**3**) as a model (Fig. 7 ▶). Although the crystal structure of (**3**) has a short axis of 4.1113 (9) Å, it is not layered like the β-structures, and the orthogonal directions are dictated by stronger interactions like O—H⋯O and Br⋯O, unlike (**1**) and (**2**). These differences are manifested in the morphology of the crystals. Unlike (**2**) which crystallizes as needles, phenol (**3**) forms plate-like crystals. In effect, the preference of Br towards type II and the change in the substitution pattern on the aromatic ring leads to a change in the mechanical property from elastic in (**2**) to brittle in (**3**).

### 3.5. Chloro/bromo exchange: a new insight   

Although Cl/CH_3_ isostructurality has been thoroughly studied over the years (Jones *et al.*, 1983 ▶; Desiraju & Sarma, 1986 ▶), and more recently with the CSD (Jones *et al.*, 1983 ▶; Desiraju & Sarma, 1986 ▶; Edwards *et al.*, 2001 ▶, 2006 ▶; Polito *et al.*, 2008 ▶; Braga *et al.*, 2009 ▶; Singh *et al.*, 2011 ▶; Nath & Nangia, 2012 ▶), Cl/Br isostructurality has not been well studied. It has been noted that Cl/Br isostructurality could arise when the Cl⋯Cl (or Br⋯Br) contact is important in the respective structures (Pedireddi *et al.*, 1992 ▶). To our knowledge, no study, with the CSD (Ouvrard *et al.*, 2003 ▶) or otherwise, has been made so far to analyze the contacts in isostructural Cl/Br compounds from the perspective of type I *versus* type II contacts. In particular, we were interested in isomorphous crystal structures in which a Cl⋯Cl interaction in one structure is replaced by a Br⋯Br interaction in another, with no other significant difference.

There are 1867 pairs of Cl- and Br-containing molecules in the CSD, in which the molecular scaffold has a Cl/Br replacement. Among them, 1060 pairs were manually selected in which the space group match and isostructurality is unambiguous. Of these, 152 contain *X*⋯*X* interactions (*X* = halogen) and a further 95 are isostructural with regard to Cl⋯Cl and Br⋯Br replacement. §2.5 gives more details. The fact that only around 15% of the 1060 pairs contain *X*⋯*X* interactions shows that these interactions are inherently weak. The other 85% or so structure pairs do not contain *X*⋯*X* interactions and are thus beyond the scope of the present study. The analysis of the 95 isostructural pairs shows that 64 cases (67.4%) have type I Cl⋯Cl and Br⋯Br interactions, and 31 (32.6%) have type II. It is instructive to examine how these preferences compare with the type I *versus* type II preference of all *X*⋯*X* contacts in the CSD. In particular, we are concerned with the type I preference of a Br⋯Br contact and the type II preference of a Cl⋯Cl contact. The analysis shows that type II C—Cl⋯Cl—C comprises 41.6% of the total C—Cl⋯Cl—C contacts in the global sample, whereas for C—Br⋯Br—C, type I contacts exist in 42.5% of the cases. The next step is to assess the propensity of formation of these (not so favourable) contacts for both the halogens in the isostructural compounds. We see that the tendency for the formation of type II contacts by Cl diminishes considerably (41.6% to 32.6%) in moving from the global sample to the limited set of 95 isomorphous pairs. The behaviour of Br is more dramatic and in a reverse sense; in the global sample 42.5% of the Br structures have a type I contact, but in the isostructural subset this rises to 67.4%. This is very significant and clearly reveals that Cl/Br isostructurality is a consequence of shape/size matching and not because of any chemical similarity between Cl and Br. The chemical character of the Cl⋯Cl contact in a general sense is also seen, because if such a character were absent, there would not be such a sizable difference in the type I/type II preference between the global set and the subset of 95 structures. Put in another way, isostructurality can arise from chemical or geometrical similarities of Cl and Br. If the reason for Cl/Br isostructurality were chemical, then the proportion of type II Cl⋯Cl contacts in the isostructural pairs would have been higher than the global value of 41.6%. In reality, it is much lower. In the end, both Cl and Br ‘modify’ themselves away from their global preferences towards a situation in which size/shape preferences dominate. Br (uncharacteristically) assumes a space-filling role in the isostructural pairs, and Cl/Br isostructurality arises because of close packing, very much like chloro/methyl exchange. Among the 152 pairs, we even found a case (YEJTUC, QOQTUL) in which Cl/Me isostructurality in one compound is replaced by Br/Me isostructurality in the other. To reinforce, Br, which normally prefers type II, is rather forced to form type I contacts which originate mainly from the geometrical model for halogen contacts, in the isostructural pairs. Even Cl behaves like this, but to a lesser extent. Isostructurality arises from the importance of the type I contacts and, in a broader sense, from the geometrical model.

It is of further interest to examine briefly the 57 pairs of compounds, from the 152 pairs originally considered, which contain Cl⋯Cl (or Br⋯Br) interactions but which were not taken into account in the above analysis. Some of these were excluded for routine reasons (see §2.5), but there is an interesting group of compounds here where a Cl⋯Cl interaction in one of the pair (LICBAA) is replaced by a Cl⋯Br interaction in the other (LIBZUR), thus formally satisfying the requirement of Cl/Br isostructurality. Similarly, there are a few pairs in which a Br⋯Br interaction in one of the pair (POWQEY) is replaced by Cl⋯Br in the other (NORLIQ). It is to be noted that in *all* these cases, the *X*⋯*X* interaction is type II and not type I. This shows definitely the chemical (rather than geometrical) nature of the Cl⋯Br interaction and confirms the electrophile–nucleophile model for the type II interaction. The value of manual intervention in computer-based CSD analyses is further underlined. If the 57 pairs of compounds above had been routinely included in the type I *versus* type II analysis, the conclusions one might have drawn could have been different. In contrast, the information that has now been gleaned about the Cl⋯Br interactions definitely adds to the overall clarity that is obtained regarding halogen atom interactions.

## 4. Conclusions   

The halogen bond continues to draw the attention of crystal engineers because of the scope it offers for design and applications. 3,4-Dichlorophenol, with its tetragonal *I*4_1_/*a* structure, is a unique and interesting compound to study halogen bonding because it has both type I and type II Cl⋯Cl interactions. Various related issues in crystal engineering have been examined using this compound as a template. 4-Bromo-3-chlorophenol is isomorphous to 3,4-dichlorophenol but 3-bromo-4-chlorophenol is not and takes a monoclinic packing. This observation owes to the fact that the type I interaction is preferred for Cl⋯Cl while type II is the choice for Br⋯Br. Variable temperature crystallography is shown to be a new and potentially reliable way of distinguishing between type I and type II halogen interactions in that the variations in contact length with changes in temperature are greater for the type II interactions. This could be due to their electrostatic nature. The alternative unobserved structures, namely the monoclinic structure for 4-bromo-3-chlorophenol and the tetragonal one for 3-bromo-4-chlorophenol, may be examined by using the CSP protocols. CSP can be used to investigate the structural landscape, and thereby complements the experimental results. Br is more electrophilic than Cl because of its greater size and polarizability, and this difference may be used as a probe to explore the fundamental difference between type I and type II *X*⋯*X* contacts. These results have been used to explain the mechanical properties of these crystalline phenols; for the first time it has been shown that plastic and elastic deformation in molecular crystals can be explained on the basis of halogen bonding. Cl/Br isostructurality is probed with reference to type I and type II preferences of Cl and Br, and reveals that this isostructurality is based on similarity in shape and size. Overall, this work summarizes a venture in modern crystal engineering in that structural insights are obtained for a class of compounds and thereafter applied to rationalize crystal properties. The correlations between halogen⋯halogen interactions in these crystals with their mechanical behaviour may be considered as an initial step towards the future design of molecular solids which will display elastic deformation upon application of stress.

## Supplementary Material

Crystal structure: contains datablock(s) 1_150K, 1_200K, 1_296K, 2_150K, 2_200K, 2_296K, 3, 4_150K, 4_200K, 4_296K, 5, TCSA_150K, TCSA_200K, TCSA_296K, 4_CBA_150K, 4_CBA_200K, 4_CBA_296K. DOI: 10.1107/S2052252513025657/bi5001sup1.cif

Structure factors: contains datablock(s) 1_150K. DOI: 10.1107/S2052252513025657/bi50011_150Ksup2.fcf

Structure factors: contains datablock(s) 1_200K. DOI: 10.1107/S2052252513025657/bi50011_200Ksup3.fcf

Structure factors: contains datablock(s) 1_296K. DOI: 10.1107/S2052252513025657/bi50011_296Ksup4.fcf

Structure factors: contains datablock(s) 2_150K. DOI: 10.1107/S2052252513025657/bi50012_150Ksup5.fcf

Structure factors: contains datablock(s) 2_200K. DOI: 10.1107/S2052252513025657/bi50012_200Ksup6.fcf

Structure factors: contains datablock(s) 2_296K. DOI: 10.1107/S2052252513025657/bi50012_296Ksup7.fcf

Structure factors: contains datablock(s) 3. DOI: 10.1107/S2052252513025657/bi50013sup8.fcf

Structure factors: contains datablock(s) 4_150K. DOI: 10.1107/S2052252513025657/bi50014_150Ksup9.fcf

Structure factors: contains datablock(s) 4_200K. DOI: 10.1107/S2052252513025657/bi50014_200Ksup10.fcf

Structure factors: contains datablock(s) 4_296K. DOI: 10.1107/S2052252513025657/bi50014_296Ksup11.fcf

Structure factors: contains datablock(s) 5. DOI: 10.1107/S2052252513025657/bi50015sup12.fcf

Structure factors: contains datablock(s) TCSA_150K. DOI: 10.1107/S2052252513025657/bi5001TCSA_150Ksup13.fcf

Structure factors: contains datablock(s) TCSA_200K. DOI: 10.1107/S2052252513025657/bi5001TCSA_200Ksup14.fcf

Structure factors: contains datablock(s) TCSA_296K. DOI: 10.1107/S2052252513025657/bi5001TCSA_296Ksup15.fcf

Structure factors: contains datablock(s) 4-CBA_150K. DOI: 10.1107/S2052252513025657/bi50014_CBA_150Ksup16.fcf

Structure factors: contains datablock(s) 4_CBA_200K. DOI: 10.1107/S2052252513025657/bi50014_CBA_200Ksup17.fcf

Structure factors: contains datablock(s) 4_CBA_296K. DOI: 10.1107/S2052252513025657/bi50014_CBA_296Ksup18.fcf

Additional supporting information. DOI: 10.1107/S2052252513025657/bi5001sup19.pdf

Elasticity in crystal 2 – movie 1. DOI: 10.1107/S2052252513025657/bi5001sup20.mov

Elasticity in crystal 2 – movie 2. DOI: 10.1107/S2052252513025657/bi5001sup21.mov

CCDC references: 938691, 938692, 938693, 938694, 938695, 938696, 938697, 938698, 938699, 938700, 938701, 959136, 959137, 959138, 959139, 959140, 959141

## Figures and Tables

**Table 1:** Crystallographic details

Name	3,4-Dichlorophenol (**1**)	4-Bromo-3-chlorophenol (**2**)	3-Bromo-4-chlorophenol (**3**)	4-Chloro-3-iodophenol (**4**)	3,5-Dibromophenol (**5**)
Chemical formula	C_6_H_4_Cl_2_O	C_6_H_4_BrClO	C_6_H_4_BrClO	C_6_H_4_ClIO	C_6_H_4_Br_2_O
Molecular weight	162.99	207.45	207.45	254.44	251.89
Crystal system	Tetragonal	Tetragonal	Monoclinic	Monoclinic	Monoclinic
Space group	*I*4_1_/*a*	*I*4_1_/*a*	*P*2_1_/*c*	*P*2_1_/*c*	*P*2_1_/*c*
*a* (Å)	26.127 (9)	26.419 (5)	11.457 (3)	11.222 (18)	11.169 (2)
*b* (Å)	26.127 (9)	26.419 (5)	4.1113 (9)	4.263 (7)	4.2067 (8)
*c* (Å)	3.7926 (9)	3.8824 (6)	15.233 (4)	15.81 (3)	14.911 (3)
α (°)	90	90	90	90	90
β (°)	90	90	108.905 (8)	106.933 (19)	91.070 (6)
γ (°)	90	90	90	90	90
Volume (Å^3^)	2588.9 (14)	2709.8 (9)	678.8 (3)	723 (2)	700.4 (2)
*Z*	16	16	4	4	4
ρ_calc_ (g cm^−3^)	1.673	2.034	2.030	2.336	2.389
*F*(000)	1312	1600	400	472	472
μ (Mo *K*α) (mm^−1^)	0.902	6.366	6.353	4.707	11.481
Temperature (K)	150	150	150	150	150
θ range for data collection (°)	3.1–27.5	3.1–27.5	3.6–27.5	2.6–27.5	3.3–27.5
*R*_1_	0.0409	0.0319	0.0360	0.0305	0.0479
*wR*_2_	0.0956	0.0623	0.0891	0.1095	0.1039
Goodness-of-fit	1.08	1.15	1.08	1.04	1.07
Reflections collected	9554	12 503	6341	7191	4635
Unique reflections	1453	1544	1557	1667	1605
Observed reflections	1178	1310	1316	1364	1227
CCDC No.	938691-3	938694-6	938700	938697-9	938701

**Table 2:** Variable temperature study of (**1**), (**2**) and (**4**) All percent increases are with respect to the values at 150 K.

		3,4-Dichlorophenol (**1**)	4-Bromo-3-chlorophenol (**2**)	4-Chloro-3-iodophenol (**4**)
150 K	Type I Cl⋯Cl	3.235 (1)	3.241 (1)	3.414 (5)
	Type II Cl⋯Cl	3.408 (1)	–	–
	Type II Br⋯Br	–	3.5379 (7)	–
	Type II 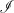 ⋯O	–	–	3.162 (6)
	Cell volume	2588.9	2709.8	723
200 K	Type I Cl⋯Cl	3.248 (1)	3.248 (2)	3.437 (4)
	% increase	0.4	0.2	0.6
	Type II Cl⋯Cl	3.431 (1)	–	–
	% increase	0.7	–	–
	Type II Br⋯Br	–	3.5580 (7)	–
	% increase	–	0.6	–
	Type II 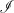 ⋯O	–	–	3.185 (5)
	% increase	–	–	0.7
	Cell volume	2615.6	2732.7	731
	% increase	1.0	0.8	1.0
296 K	Type I Cl⋯Cl	3.268 (1)	3.278 (3)	3.453 (5)
	% increase	1.0	1.1	1.1
	Type II Cl⋯Cl	3.469(2)	–	–
	% increase	1.8	–	–
	Type II Br⋯Br	–	3.613 (3)	–
	% increase	–	2.1	–
	Type II 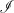 ⋯O	–	–	3.214 (6)
	% increase	–	–	1.6
	Cell volume	2654.7	2817	740
	% increase	2.5	3.9	2.3
