# Supplementary material for: Halogen bonds in some dihalogenated phenols: applications to crystal engineering
Source: IUCrJ. 2013 Oct 18;1(Pt 1):49–60. doi: 10.1107/S2052252513025657 (PMC4104968; doi:10.1107/S2052252513025657)
Supplement: Supplementary file 19 [file m-01-00049-sup19.pdf]

Halogen bonds in some dihalogenated phenols. Applications to crystal engineering.

Arijit Mukherjee and Gautam R. Desiraju*

Solid State and Structural Chemistry Unit, Indian Institute of Science, Bangalore, India

Fax: +91 80 23602306; Tel: +91 80 22933311; E-mail: desiraju@sscu.iisc.ernet.in

Supporting Information

Contents

**S1.**	Computational details	**p. 2**
**S2.**	Database Studies	**p. 3**
**S3.**	Hydrogen bond table	**p. 4**
**S4.**	ORTEP diagrams	**p.5**
**S5.**	Video showing the elasticity of **2**	**p.6**
**S6.**	Crystal structure of 3,5-dibromophenol (5)	**p.7**
**S7.**	Crystallographic table for 4-chlorobenzoic acid and 2,3,5-trichlorosalicylic acid	**p.7**
**S7.**	Results of variable temperature study performed on 4-chlorobenzoic acid and 2,3,5-trichlorosalicylic acid.	**p.8**

S1. Computational Methodology

The experimental structure of 3,4-dichlorophenol at –123°C was taken as an input in the crystal structure prediction (CSP) of **1**,**2** and **3**. The structure was then optimized and ESP charges were assigned in DMol3 component of Materials Studio. Optimized structures were taken as input structures in CSP protocol. For **2** and **3**, the same procedure of optimization was followed after respective substitutions. The CSP protocol has been detailed below:

Protocol: Packing >> Clustering >> Geometry Optimization >> Clustering
Version: 6.0

---- Packing parameters ----

Search algorithm	:	MC Simulated Annealing
Maximum number of steps	:	7000
Explore torsions	:	No
Pre-optimize structures	:	No
Steps to accept before cooling	:	20
Minimum move factor	:	0.1000E–06
Heating factor	:	0.02500
Maximum temperature	:	100000.0 K
Minimum temperature	:	300.0 K

---- Cluster analysis parameters ----

Cluster grouping	:	Forcefield type
Cutoff	:	7.000
Number of bins	:	140
Tolerance	:	0.2000
Maximum number of clusters	:	All clusters

---- Geometry optimization parameters ----

Algorithm	:	Smart
Convergence tolerance:		
Energy	:	0.001 kcal/mol
Force	:	0.5 kcal/mol/A
Maximum number of iterations	:	500
External pressure	:	0 GPa
Motion groups rigid	:	YES
Optimize cell	:	YES

---- Energy parameters ----

Forcefield	:	COMPASS26

Electrostatic terms:

Summation method	:	Ewald
Accuracy	:	0.001 kcal/mol
Buffer width	:	0.5 A

van der Waals terms:

Summation method	:	Ewald
Accuracy	:	0.001 kcal/mol
Repulsive cutoff	:	6 Å
Buffer width	:	0.5 Å

S2. Database Studies: Cl/Br isostructurality

A. Type I: 64 pairs (67.4%)

RIGLID<br>RIGLOS	RUFGOP<br>VIDFEU	ONEDAR<br>ONEDIV	VEJGUN<br>VEJHAV	JEBGAZ<br>AFEQIM
CLANTO<br>BRANTO	IFULUQ04<br>WASHEE	NAGVUM<br>OHIJAR01	IWOXIB<br>XEHOL	CLURAC10<br>BRURAC11
NUYJUM<br>NUYKAT	MUTBEJ<br>MUTBOT	MEHGAI<br>GEPNEV	XICPEF<br>QOFKIG	ZZVTY12<br>TPHMBR02
QEDXEC<br>YIHZAQ	HALDOL01<br>BIBSEK	OLOHIG<br>ROFBIY	SULTOJ<br>SULTUP	TAQBUJ<br>TAQCAQ
KAJFUX<br>KAJFIL	XUBPIT<br>XUBPOZ	DUPTOY<br>FEYVAH	HOFKET<br>KUSNIW	ULUPIA<br>JAMXEB
SEHHIX02<br>SAZZAV	TIKTOX<br>TIKTIR	WOGYAT<br>DUDCUB	QOFKAY<br>PIDROK	MOSWAT<br>SOYTUW
OGEKUH<br>WADGAL	XOBKEE<br>XOBKII	HUMHUS<br>YIZRAB	QALNOH<br>QALNUN	LEMLEV<br>LEMLIZ
ADALUN<br>RODGIA	PUZZAM<br>UDURAN	MIGXAC<br>SEZFEJ	QERTIR<br>SEZNUH	CBZCAN01<br>MBZCLD10
TIZVUU<br>TIZWAB	NISCOI01<br>NOLYET01	SUZKUU<br>SUZZIX	KOFFIV<br>BOQWEJ	QAKBOU<br>QAKBUA
YAKWUC<br>YAKXAJ	JEPYAF<br>JEPZIO	SIRXAT<br>VASMEI	ISOBUO<br>ISOCAV	SUZGUQ<br>TUPSUT
CAVQIZ<br>ULELON	TINWET<br>MODWOS	NULROC<br>NULRUI	SAZZID<br>SAZYOI	GEPNUL<br>AFEHOJ
NULSAP<br>NULSET	JEPXUY<br>JEPZEK	SEHHET<br>SAZYUO	VOQREZ<br>HODPOG	SACTAS<br>CACSOP
NULQUH<br>NULRAO	COTMON<br>TEJSIK	COBBEA<br>CILYUF	SEFYUX<br>VITVID	

Type II: 31 pairs (32.6%)

DOZJAD<br>ZUYZAU	GACFOF<br>HEJNEP	MUVBAG<br>MUTZUW	COXNEI<br>BRGUOS01	CBALOS01<br>BAGWOW
YICFAR	GAKNAH	CLDOUR	BAJVUD	HECKOP

YICFEV	IJUXEQ01	BROXUR11	YUNWOU	YUHZAC
XOXKAX<br>XOXKEB	BNQDCP<br>BNQDBP	MECINT<br>MBRCIN	MOKMIJ<br>CUDDEL	IGEHEI<br>IGEHIM
CPHACR<br>BPHACR	TIHZOA<br>TIHZEQ	DIRNOH<br>TOHTUG	ODEPUJ<br>BPCBZS11	DUPFUQ<br>PUJKIP
NABRAJ<br>BRBZAM	RALNEY<br>HEWRIL	OFIWAB<br>OFIWEF	AJETOZ<br>DUJJOI	YAYVIE<br>YAYVUQ
OMEGUJ<br>OMEHAQ	TIHYOZ<br>TIHYEP	TIHWUD<br>BPCBZS11	AMCLPY<br>CAJXAN	SETGII<br>HIRQIJ
NEBFAB<br>NEBFEF				

S3. Hydrogen bond table

All distances obtained at –123 °C are neutron normalized.

	D–H···A	Symmetry code	D–H (Å)	H···A (Å)	D–H···A (°)
3,4-Dichlorophenol (**1**)	O1–H1O···O1	7/4-y,1/4+x,1/4+z	0.98	1.74	171
4-Bromo-3-chlorophenol (**2**)	O1–H1O···O1	1/4+y,5/4-x,1/4+z	0.98	1.76	169
3-Bromo-4-chlorophenol (**3**)	O1–H1O···O1	1-x,1/2+y,-1/2-z	0.98	1.69	170
4-Chloro-3-iodophenol (**4**)	O1–H1O···O1	1-x,-1/2+y,3/2-z	0.98	1.73	165
3,5-Dibromophenol (**5**)	O1–H1O···O1	-1-x,1/2+y,3/2-z	0.98	1.71	176

S4. ORTEP diagrams

3,4-Dichlorophenol (1)

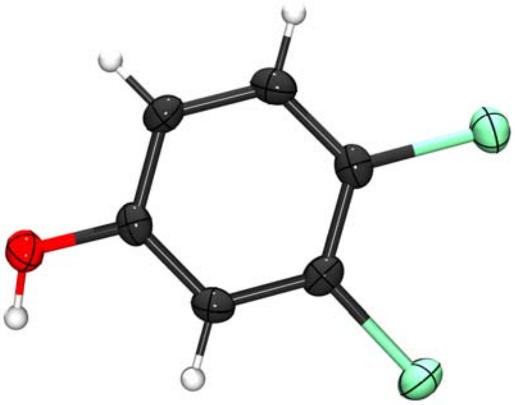

4-Bromo-3-chlorophenol (2)

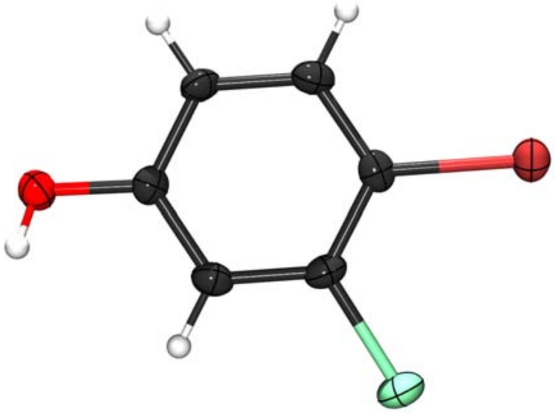

3-Bromo-4-chlorophenol (3)

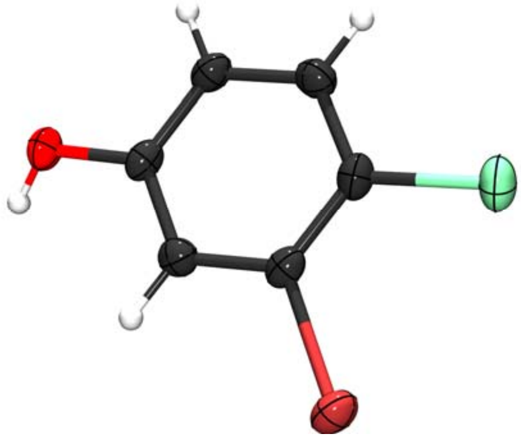

4-Chloro-3-iodophenol (4)

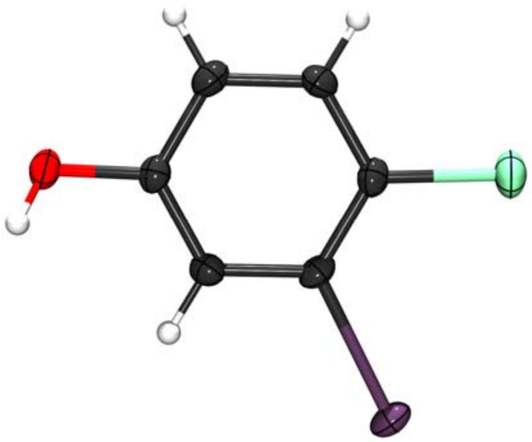

3,5-Dibromophenol (5)

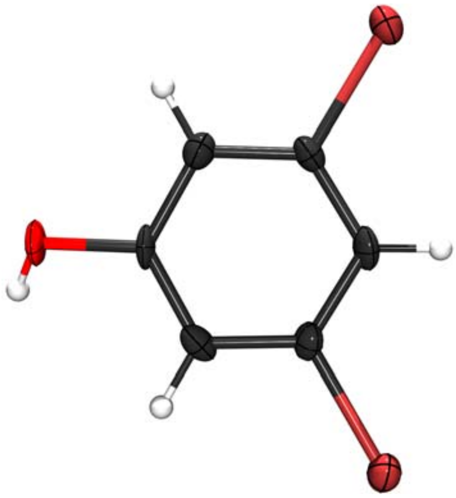

S5. Video showing elastic deformation in 2

Videos are provided as separate files.

S6. Crystal structure of 3,5-dibromophenol (5)

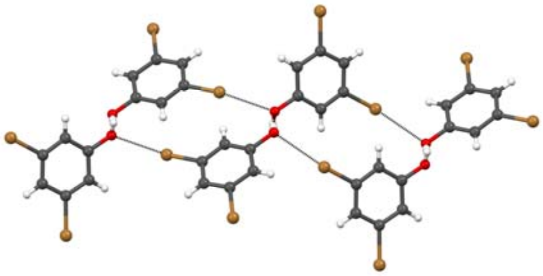

S7. Crystallographic table for 4-chlorobenzoic acid and 2,3,5-trichlorosalicylic acid

Name	4-Chlorobenzoic acid	2,3,5-Trichlorosalicylic acid
Formula	$C_7H_5ClO_2$	$C_7H_3Cl_3O_3$
Molecular weight	156.56	241.44
Crystal system	Triclinic	Monoclinic
Space group	*P*-1	$P2_1/c$
a (Å)	3.8017(8)	4.9531(14)
b (Å)	6.1607(12)	24.020(6)
c (Å)	14.208(3)	8.007(3)
α (°)	92.417(7)	90
β (°)	94.718(7)	118.88
γ (°)	92.286(7)	90
Volume ($Å^3$)	331.03(12)	834.2(5)
Z	2	4
ρ_{calc} (g/cm^3)	1.571	1.923
F(000)	160	480
μ (MoK_α) (mm^{-1})	0.499	1.062
Temp. (K)	150K	150K
θ Range for data collection (°)	3.3, 27.5	1.7, 27.6
R_1	0.0347	0.0468
wR_2	0.0977	0.1452
Goodness-of-fit	1.11	1.11
Reflns collected	3353	5589
Unique reflns	1496	1904
Observed reflns	1356	1764
CCDC No.	959136-959138	959139-959141

S8. Results of variable temperature study performed on 4-chlorobenzoic acid and 2,3,5-trichlorosalicylic acid.

		4-Chlorobenzoic acid	2,3,5-trichlorsalicylic acid
–123 °C	Type I Cl···Cl	3.4095(8)	
	Type II Cl···Cl		3.473(1)
	Cell Volume	331.031	834.146
–73 °C	Type I Cl···Cl	3.4186(8)	
	% increase	0.27%	
	Type II Cl···Cl		3.498(1)
	% increase		0.72%
	Cell Volume	333.631	843.821
	% increase	0.79%	1.16%
23°C	Type I Cl···Cl	3.448(1)	
	% increase	1.13%	
	Type II Cl···Cl		3.547(1)
	% increase		2.13%
	Cell Volume	341.16	855.868
	% increase	3.05%	2.6%
